# Correlation of Oxidative Stress with Serum Trace Element Levels and Antioxidant Enzyme Status in Beta Thalassemia Major Patients: A Review of the Literature

**DOI:** 10.1155/2012/270923

**Published:** 2012-05-09

**Authors:** Q. Shazia, Z. H. Mohammad, Taibur Rahman, Hossain Uddin Shekhar

**Affiliations:** ^1^School of Medicine, Universiti Malaysia Sabah (UMS), Locked Bag 2073, 88999 Kota Kinabalu, Sabah, Malaysia; ^2^Department of Biochemistry and Molecular Biology, University of Dhaka, Dhaka 1000, Bangladesh

## Abstract

Beta thalassemia major is an inherited disease resulting from reduction or total lack of beta globin chains. Patients with this disease need repeated blood transfusion for survival. This may cause oxidative stress and tissue injury due to iron overload, altered antioxidant enzymes, and other essential trace element levels. The aim of this review is to scrutinize the relationship between oxidative stress and serum trace elements, degree of damage caused by oxidative stress, and the role of antioxidant enzymes in beta thalassemia major patients. The findings indicate that oxidative stress in patients with beta thalassemia major is mainly caused by tissue injury due to over production of free radical by secondary iron overload, alteration in serum trace elements and antioxidant enzymes level. The role of trace elements like selenium, copper, iron, and zinc in beta thalassemia major patients reveals a significant change of these trace elements. Studies published on the status of antioxidant enzymes like catalase, superoxide dismutase, glutathione, and glutathione S-transferase in beta thalassemia patients also showed variable results. The administration of selective antioxidants along with essential trace elements and minerals to reduce the extent of oxidative damage and related complications in beta thalassemia major still need further evaluation.

## 1. Introduction

Beta thalassemia is one of the most common inherited single gene disorder caused by about 200 mutations in the beta globin genes. In beta thalassemia where there is no or reduced production of beta globin chains, the alpha chain production will continue to occur. This increased synthesis of alpha chains makes the developing erythrocytes more fragile leading to early damage, ineffective erythropoeisis and anemia. Beta thalassemia exists in different forms depending upon the beta globin chains deficit. The most severe form among them is beta thalassemia major which occurs as a result of inheritance of two beta globin chain mutations either in homozygous or compound heterozygous states. Patients with beta thalassemia major need repeated blood transfusions for survival due to severe anemia. The beta globin chain deficit for beta-thalassemia trait (minor) is 50%, while that for beta-thalassemia major is 100% and between 50–80% for beta-thalassemia intermediate [1]. Malaysia has a multiracial population of 27.7 million, consisting of 50.8% Malays, 23.0% Chinese, and 6.9% Indians, indigenous people of Sabah and Sarawak (11.0%), and other minority groups (8.3%) [2]. According to the report of Malaysian Ministry of Health, one (1) out of twenty (20) Malaysians is a carrier of thalassemia. In Malaysia, there are about 600,000–1 (one) million thalassemia carriers. They are common among Malays, Chinese, and Sabahans but rarely among Indians and native Sarawakians. Thalassemia major is considered to be one of the life-threatening genetic disorders in Malaysia with the gene frequency of 3.4–4.5% [3]. Recurrent blood transfusions in beta thalassemia major lead to accumulation of excess iron in the body tissues. This secondary iron overload is responsible for peroxidative damage by increased production of reactive oxygen species within the erythrocytes leading to oxidative stress. This oxidative stress will cause growth failure as well as liver, cardiovascular, endocrine, and neurological complications in beta thalassemia major. It has been evident from previous studies that iron overload is the main causative agent responsible for increased production of free radical and reactive oxygen species and subsequent oxidative stress which is compensated by various antioxidants present in the body. These antioxidants are complex molecules that protect important biological sites from oxidative injury [4] in a retrospective study involving 123 thalassemia major children found that the most common complication among these beta thalassemic children was growth failure (57.8%) which may be due to neurosecretary disturbance and insensitivity of growth hormone. They further concluded that chronic anemia and hemosiderosis may also be the contributing factors to growth failure. The next is the liver problems (21.1%), heart diseases (13.8%), and endocrinopathies (4.2%).

## 2. Oxidative Stress

Oxidative stress is defined as the interruption of balance between oxidants and reductants within the body due to the excess production of peroxides and free radicals. This imbalance will cause damage to cellular components and tissues in the body leading to oxidative stress. In patients with beta thalassemia major where frequent blood transfusions are required due to severe anemia, oxidative stress occurs as a result of increased levels of lipid peroxides and free-radical intermediates, as well as the decrease in total antioxidant capacity. Use of iron chelatory agents in combination with antioxidants can be helpful in the regulation of the antioxidant status in patients with beta thalassemia major. Oxidative stress and disturbance in antioxidant balance in beta thalassemia major has been studied extensively [5, 6]. Seventy-two children with beta thalassemia major on iron chelation therapy and 72 age-matched healthy controls irrespective of sex were included in the study. They found a significant increase in the levels of lipid peroxide and iron and significant decrease in levels of vit E and total antioxidant capacity. Serum zinc was significantly increased while copper levels decreased and there is a nonsignificant increase in erythrocyte superoxide dismutase. The results suggested that the oxidative stress and decreased antioxidant defence mechanism play an important role in the pathogenesis of beta thalassemia major.

It is concluded that repeated blood transfusions in beta thalassemia major patients causes secondary iron overload and this makes erythrocytes vulnerable to peroxidative injury [7]. Iron overload leads to peroxidative damage in beta-thalassemia major and antioxidant systems try to reduce tissue damage by lowering lipid peroxidation. They found that the markers of lipid peroxide damage such as melon-dialdehyde, antioxidant enzyme superoxide dismutase, and nitric oxide levels were significantly raised in thalassemia major children while mean glutathione peroxidase (GPx) levels were reduced in patients as compared to controls. These markers significantly correlated with serum ferritin levels. There was no significant difference in Glutathione (GSH) levels but it correlated with serum iron levels.

## 3. Oxidative Stress and Serum Trace Elements

Trace elements and the minerals play a vital role in the body to perform its functions properly. These elements and the minerals should present in the body in appropriate amounts and must be available for reacting with other elements to form critical molecules as well as to participate in various important chemical reactions. A number of trace elements are found in human plasma and here we are interested to discuss the correlation of trace elements like selenium, copper, zinc, and iron with oxidative stress in beta thalassemia major.

### 3.1. Selenium

One of the essential trace elements in human plasma is selenium. Selenium was first discovered as a byproduct of sulfuric acid production. It is a well-known electrometalloid and is mostly famous due to its anti cancerous properties. It is an essential constituent of the enzyme glutathione peroxidase and also incorporates in various important proteins such as hemoglobin and myoglobin. Selenium is also a component of the unusual amino acids selenocysteine which is essential for the production of various useful enzymes in the body. It helps in preventing free radical damage caused by ferrous chloride, and heme compounds. Its deficiency may affect the iron binding capacity of transferrin which leads to increase iron stores and subsequent tissue damage. An age- and gender-matched case control study has been conducted on patients with beta thalassemia major on iron chelation therapy [8]. The study indicates a significant decrease in plasma concentrations of the essential element selenium as well as decreased plasma activity of selenium-dependent antioxidant enzyme glutathione peroxidase (GPx). They also found significantly increased concentrations of all measures of body iron in beta-thalassemia patients as compared to healthy controls: another study on relationship between iron overload and antioxidant micronutrient status among 64 transfusion-dependent beta thalassemia major children on chelation therapy and 63 age- and sex- matched controls [9]. They measured serum levels of vitamins A and E, zinc, selenium, and copper and found significantly decreased levels of all these elements in beta thalassemia major children as compared to controls. There is a study done to evaluate the *in vitro* effects of vitamin C and selenium on natural killer cell activity of beta thalassemia major indicates a significant decreased in natural killer cell activity in all thalassemic patients as compared to control. The NK activity is increased by low-dose selenium treatment but no change is observed in control group. High-dose selenium decreased NK activity significantly in splenectomised patients. The result indicates the careful use of selenium dosage in thalassemic major patients [10].

### 3.2. Copper

Copper is the other essential trace element present in our bodies. It mostly forms metalloprotiens which act as enzymes. Copper is the major component of hemoglobin which is a protein responsible for oxygen transport in blood cells. Along with vitamin C, it is responsible for the production of protein called elastin thus maintaining the elasticity of the skin, blood vessels, and lungs. It is antibacterial and bears important antioxidant properties. Copper is a central component of the antioxidant superoxide dismutase molecule and also helps in the formation of protein called ceruloplasmin thereby protecting the cells from free-radical injury. Copper is also required for the production of hormones like nor adrenaline and prostaglandins which are hormone-like chemicals involved in the regulation of blood pressure, pulse, and healing. Deficiency of this trace element will lead to anemia, neutropenia, and growth impairment, abnormalities in glucose and cholesterol metabolism, and increased rate of infections. On the other hand, an accumulation of copper in body leads to Wilson's disease with copper accumulation and cirrhosis of liver. A prospective study was performed to determine the serum levels of zinc and copper in beta thalassemia major children [11]. This cross-sectional study revealed that hypozincemia is common in thalassemic patients, but there is no copper deficiency. Another study was carried out to evaluate the level of some essential elements in one hundred and five thalassemic blood-transfusion-dependent patients and 54 healthy controls [12]. They found lower serum zinc and magnesium levels and higher copper and potassium levels in thalassemic major patients as compared to controls. Zinc deficiency may be due to hyperzincuria resulted from the release of zinc from hemolyzed red cells while hypercupremia occurs in acute and chronic infections and hemochromatosis which is the principal complication of thalassemia. A study done on status of thyroid function and iron overload in patients with beta thalassemia major on Deferoxamine in Jordan concluded that there is significantly high (*P* < 0.05) levels of serum ferritin, FT3, zinc, and copper in patients with beta thalassemia major as compared to controls [13].

### 3.3. Zinc

The next essential trace element present in the body is zinc. It takes part in various important body functions including protein synthesis, DNA synthesis, and cellular growth. It is found almost in every cell and plays a vital role in body's immune system affecting innate and acquired immunity. Zinc also has significant antioxidant properties thereby protecting the cells from damage due to free radicals. It is the active site for a number of metalloenzymes which are required for nucleic acid synthesis and also important for other host defense mechanisms like production of monocytes and macrophages and chemotaxis of granulocytes [14]. Zinc is absorbed from small intestine and found in the blood bound to albumin. Impaired growths, alopecia, loss of weight are few of the associated complications due to deficiency of zinc which is one of the factors responsible for growth and puberty disorders in thalassemic patients [15]. Frequent blood transfusions can lead to iron overload which may result in various endocrine abnormalities. They have studied two hundred twenty patients with beta thalassemia major on chelation therapy. They found that there is an association between the duration of chelation therapy and abnormalities in lumbar bone mineral density (BMD). Low serum zinc and copper was observed in 79.6% and 68% of the study population, respectively. There is significant association of serum zinc levels with lumbar but not femoral BMD. Another study was carried out to evaluate the serum copper and zinc in Jordanian thalassaemic patients. Forty two patients with *β*-thalassemia major on periodical blood transfusion and Deferoxamine were included in this study [16]. Forty age- and gender-matched healthy controls were included in the study. The results indicate that copper and zinc levels were significantly increased in beta thalassemia major patients compared with controls. These finding may be explained by the decreasing rate of glomerular filtration of zinc seen in chronic hemolysis and the disturbance in the metabolism of zinc and copper in thalassaemic patients due to the increasing serum zinc. The high level of copper could be due to increase absorption of copper from gastrointestinal tract. It is shown that a case control prospective study including 100 beta thalassemia major patients with heights within 3rd to 10th percentile [17]. They randomly divide patients in two groups ach comprising of 50 patients. Group 1 was given oral zinc supplements while group 2 is a control group with no zinc supplements. The patients were observed for 18 months. They found out that there is no significant difference in height between the two groups after 18 months of observation and concluded that oral zinc sulphate has no significant effect on linear growth of beta thalassemia major patient.

### 3.4. Iron

Iron is another essential trace element present in almost all cells of the body. Human body requires iron for the synthesis of oxygen carrying protein called haemoglobin found in red blood cells, and myoglobin which is also a protein found in muscles. It also takes part in the production of other important proteins in the body such as for DNA synthesis and cell division. Furthermore, iron is used in the connective tissues in our body, some of the neurotransmitters in our brain, and to maintain the immune system. Iron is transported through the blood by the serum protein, called transferrin. Transferrin is normally 30% saturated with iron. The total iron-binding capacity (tibc) reflects the status of iron in the body and is defined as the amount of iron needed for 100% transferrin saturation. The levels of TIBC are raised when the levels of iron are low thus will be helpful in the diagnosis and monitoring of iron deficiency anaemia. When iron is present in excess amounts in the body it will lead to hemochromatosis, which may be primary or secondary. Primary hemochromatosis is a genetic disorder characterized by increased iron absorption and consequent iron overload in the body. Secondary hemochromatosis occurs in diseases like thalassemia due to iron overload especially in thalassemia major where repeated blood transfusions are required. Beta thalassemia major patients require frequent blood transfusions which lead to iron overload in the absence of effective chelation therapy. This iron deposits in thalassemic patients can exceed from the storage and detoxification capacity of ferritin and also fully saturates transferrin and leads to the formation of free iron which accumulates in blood and tissues. This free iron will cause the formation of very harmful compounds, such as hydroxyl radical (OH). The hydroxyl radicals are highly reactive and attacks lipids to form lipid peroxides which contribute to oxidative stress [18]. Regular blood transfusions along with chelation therapy in beta thalassemia patients drastically improve the quality and duration of life to third and fourth decades. Iron overload is serious complication of long-term blood transfusion. It requires adequate treatment in thalassemics so that the early deaths especially from iron-induced cardiomyopathies will be prevented. It has been shown that the cardiovascular involvement in beta thalassemia major patients without cardiac iron overload [19]. They involved twenty six patients with beta thalassemia major on chelation therapy without cardiac iron overload and thirty age- and gender-matched healthy controls in the study. The results indicated aortic stiffening associated with increased left ventricular mass and left atrial enlargement in the beta thalassemia patients as compared to controls. These changes may represent the signs of early cardiovascular involvement in beta thalassemia patients without cardiac iron overload. A retrospective chart view study revealed that three hundred and sixty transfusion-dependent beta thalassemic patients treated with Deferoxamine [20]. All patients were followed and treated from 1990–2004, disease complications were assessed by the measuring iron overload and mean serum ferritin concentrations yearly for all patients. The result showed that cardiac complications being the first most important cause of death followed by infections. Complications and deaths among these beta thalassemic major patients is iron-related organ dysfunction and age related. Furthermore, serum ferritin levels were found significantly higher in patients who died as compared to those who survived. It was also found that majority of the complicated patients were on nonoptimal chelation therapy and noncompliance. Early detection of iron overload on the heart is crucial in the management of beta thalassemia major. Serum ferritin is the poor indicator of myocardial iron deposition during early iron overload stage [21].

### 3.5. Magnesium

Magnesium is another trace element which is essential for maintaining proper body functions. It is vital for body's immune system, cardiovascular, and musculoskeletal systems. Deficiency of this element will lead to hypertension, diabetes, and cardiovascular diseases. A study was carried out to evaluate the level of some essential elements in one hundred and five thalassemic blood-transfusion-dependent patients and 54 healthy controls [12]. They found lower serum zinc and magnesium levels and higher copper and potassium levels in thalassemic major patients as compared to controls. Zinc deficiency may be due to hyperzincuria resulted from the release of zinc from hemolyzed red cells while hypercupremia occurs in acute and chronic infections and hemochromatosis which is the principal complication of thalassemia.

### 3.6. Iodine

The other vital trace element present in the body is iodine and is one of the powerful antioxidants present in the body. Bernard Courtois, a French chemist, was first discovered iodine in 1811. Iodine is present in almost every body tissue but found in greater quantities in thyroid, breast, stomach, liver, lungs, heart, adrenals, and ovaries. Iodine is important for mental and physical development and maintaining healthy immune system. It takes part in the production of thyroid hormones including thyroxin and tri-iodothyronine. These hormones are of primary importance in maintaining the body metabolism and brain development. Deficiency of this trace element may lead to cancer, diabetes, heart diseases, and multiple sclerosis. A study revealed increased sensitivity to the inhibitory effect of excess iodide on thyroid functions in 25 beta thalassemia major patients with normal thyroid functions [22]. The patients were given 20 mg of iodine three times daily for three weeks. They found significant decrease in concentration of thyroid hormones and significant increase in TSH concentrations with 56% of the patients reached to hypothyroid levels. They concluded that beta thalassemia major patients should not be given excess iodide due to increased sensitivity to inhibitory effects on thyroid functions as it may lead to permanent hypothyroidism. A study was carried out on long-term intensive combined chelation therapy on thyroid function in 51 beta thalassemia major patients after they achieved negative iron balance [23]. While on Deferoxamine monotherapy, eighteen patients required thyroxin but after combined therapy with deferrioxamine and deferiprone there is significant (*P* < 0.0001) decrease in iron overload and a significant increase in mean FT4 and FT3 concentrations with mean decrease in TSH. They concluded that negative iron balance can be achieved rapidly with combination chelation therapy than with monotherapy as well as there is reversal of hypothyroidism with this regime.

### 3.7. Calcium

Calcium is one of the most abundant trace elements present in the body. It is important for regulating cardiovascular, musculoskeletal, and nervous systems of the body. Deficiency of calcium may lead to rickets, osteomalacia, and osteoporosis. Excess of this trace element may lead to kidney stones, impaired renal functions, and prostate cancer. Studies have shown that calcium may activate the enzymes involved in the production of reactive oxygen species and free radicals by the mitochondria. It has been shown that seventy-five percent of patients had a low calcium level and 72.5% of patients had hypothyroidism [24]. The low calcium level was probably caused by a combination of hypoparathyroidism and osteomalacia resulting from deficient calcium intake. A case control study done on the effects of intramuscular injection of a megadose of cholecalciferol involving 40 beta thalassemia major patients and 40 nonthalassemic controls [25]. They found that among thalassemia major patients, two had hypoparathyroidism and low 25-OH D, and two had hypocalcaemia with hypophosphatemia, high alkaline phosphatase (ALP), high PTH, and serum 25-OH D below ng/mL. The remaining patients had low 25-OH D concentrations with normal serum Ca and PO4 concentrations. Vitamin D deficiency is present in 100% of thalassemia major patients and treatment with megadose injection of cholecalciferol is effective for hypovitaminosis D for 3 months. A case study done on 14-year-old girl with beta thalassemia major diagnosed since the age of 9 months came to their center with generalized tonic clonic seizure [26]. The investigations revealed diffuse intracranial calcifications in deep white matter, posterior fossa, basal ganglia, and both thalami. The laboratory and neuroimaging also indicate hypoparathyroidism. They recommend periodic assessment and control of serum calcium in all patients with thalassemia major and prompt treatment with oral calcium and active form of vitamin D can prevent hypoparathyroidism and neurological complications in beta thalassemia major patients.

## 4. Oxidative Stress and Antioxidant Enzymes

 Oxidative stress in beta thalassemia major patients activates various antioxidant enzyme systems to protect the body tissues from its damaging effects. A large number of antioxidant enzymes present in the body, here we are interested to determine the antioxidant status of the following enzymes in beta thalassemia major:

- superoxide dismutase,
- glutathione peroxidase (GPx),
- glutathione (GSH),
- glutathione S transferase,
- catalase.

### 4.1. Superoxide Dismutase

One of the most important antioxidant enzymes present in the human body is superoxide dismutase. It exists in several different forms and was first discovered by two biochemist named Irwin Fridovich and Joe McCord. Superoxide dismutases are the proteins cofactor with copper, zinc manganese, iron, or nickel. In humans, it exists in three different forms including SOD1 found in cytoplasm, SOD2 present in cytoplasm, and SOD3 is extracellular. Superoxide is the main reactive oxygen species which react with nitric oxide radical and forms peroxynitrite thereby causing oxidative stress and cellular damage. SOD is the essential antioxidant that decreases the formation of reactive oxygen species and oxidative stress thus protecting the cells from damage. Erythrocyte superoxide dismutase protects the erythrocyte from being damaged during oxidative stress. A study revealed higher levels of erythrocyte superoxide dismutase and glutathione peroxidise (GPx) as well as higher plasma melanoyldialdehyde (MDA) in thalassemia major patients as compared to healthy controls [27]. They suggested that increased levels of malondialdehyde may be due iron overload through repeated blood transfusions and subsequent oxidative stress produced by reactive oxygen species. The rise in superoxide dismutase and glutathione peroxidase may occur as a result of compensatory mechanisms in response to oxidative stress.

### 4.2. Glutathione Peroxidase

Other important antioxidant enzymes found in the humans is glutathione peroxidase. It belongs to a group of antioxidant selenoenzymes that protects the cells from damage by catalyzing the reduction of lipid hydroperoxides. This action requires the presence of glutathione. Glutathione peroxidase levels in the body are in close relation with the glutathione which is the most important antioxidant present in the cytoplasm of the cells. The stability of the cellular and subcellular membranes depends mainly on glutathione peroxidase and the protective antioxidant effect of glutathione peroxidase depends on the presence of selenium. Glutathione peroxidase (GPx) also protects the heart from damage by oxidative stress due to oxygen free radicals through its antioxidant effect. A study was conducted on fifty six beta thalassemia major patients and fifty one healthy controls. The findings of the study confirm the peroxidative status generated by iron overload in beta thalassemia major patients and the significant increase in serum ferritin, iron, plasmatic thiobarbituric acid reactive substances (TBARS), and plasmatic superoxide dismutase (SOD) and glutathione peroxidase (GPx) activity, but vitamins E and zinc concentrations were significantly decreased in beta thalassemia major patients [28].

### 4.3. Glutathione (GSH)

The next vital antioxidant enzyme in the body is glutathione which is a tripeptide containing three amino acids. It is present in almost all living cells and is considered to be the most powerful and most important antioxidant produced in the human body. It prevents damage to the cellular components by reactive oxygen species including free radicals and peroxides. It also exhibits strong anticancer and antiviral properties. Glutathione is important for the protection of proteins involved in the synthesis of nucleic acid and also helps in DNA repair. It plays an important role in the body's immune function through white blood cells as well as maintains the red blood cells integrity [29]. Glutathione is found exclusively in its reduced form (GSH). The oxidized glutathione or glutathione disulphide (GSSH) is converted to its reduced sulfhydryl form (GSH) which is a potent antioxidant, by the enzyme glutathione reductase, which becomes activated upon oxidative stress. Ratio of reduced glutathione to oxidized glutathione can be used to determine the cellular toxicity. In a study, researcher analysed glutathione reductase, glucose-6-phosphate dehydrogenase, and glutathione peroxidase in twenty five cases of homozygous beta thalassaemia, twenty cases of heterozygous beta thalassaemia and ten controls. The results indicate that significant elevation of these enzymes in homozygous beta thalassemia shows the presence of enzyme regulated glutathione turnover system in the overt state to overcome the red cell membrane damage due to autooxidant threat [30].

### 4.4. Glutathione S Transferase

Glutathione S transferase belongs to the group of enzymes that catalyze a number of reactions in the body. It catalyzes the conjugation of reduced glutathione through sulphydryl group to electrophilic centres. This activity is responsible for detoxification of compounds like lipid peroxides. It has been observed that GSTM1 which is the member of glutathione S-transferase family plays an important role in detoxification of metabolites of xenobiotics involved in cancer. Homogenous deletion of this GSTM1 results in a lack GSTM1 enzyme activity and is associated with lung, bladder, prostate, and other tumors. Genetic variations of GSTM1 enzyme are associated with patients receiving regular chelation therapy [31].

### 4.5. Catalase

Catalase was first discovered by Louis Jacques Thenard in 1818. It is an intracellular enzyme made up of four polypeptide chains with four porphyrin heme groups. Catalase is responsible for detoxification of hydrogen peroxide in the cells. Alteration in gene expression of this enzyme will lead to increased risk of cancer. A study revealed increased levels of antioxidant enzymes superoxide dismutase, catalase, and glutathione peroxidase in red blood cells of beta thalassemia minor and near normal values of these enzymes in red blood cells of beta thalassemia major patients. They concluded that the red cells in beta thalassemia minor react to increased oxidant threat with augmented antioxidant enzyme activities while in beta thalassemia major patients normal antioxidant enzyme levels are due to presence of normal red cells because of to multiple blood transfusions [32].

## 5. Conclusion

This comprehensive review of literature indicates that oxidative stress in patients with beta thalassemia major is mainly caused by peroxidative injury due to secondary iron overload. Production of free radicals by iron overload, alteration in serum trace elements, and antioxidant enzymes status play an important role in the pathogenesis of beta thalassemia major. Impairment of the antioxidant status is associated with elevated plasma levels of lipid peroxidation. There is limited data available concerning oxidative stress, antioxidant status, degree of peroxidase damage, and role of trace elements in beta thalassemia major patients. Studies on trace elements like selenium, copper, iron, zinc, magnesium, iodine, and calcium reveal significant change in plasma concentration of these trace elements in beta thalassemia major patients. Zinc levels in beta thalassemia major patients were significantly decreased in most of the studies as compared to the controls. The reason proposed being hyperzincuria due to the release of zinc from hemolysed red cells. The patients suffering from beta thalasemia major do not survive for more than 5 years without blood transfusion [33]. A contrary study showed significantly reduced levels of serum zinc in beta thalassemia major patients [9]. Copper, another essential trace element, was found to be significantly decreased [9, 15] on thalassemia major patients but high levels of copper as compared to controls. This increased level of copper may be due to acute or chronic infections and hemochromatosis that occurs as complications in thalassemia major [12]. There is one prospective study indicating no change in serum copper levels in thalassemia major patients. Iron being the most important of all minerals was found to be significantly increased in beta thalassemia major patients [11]. Probably due to repeated blood transfusions and increased iron absorption from gastrointestinal tract. Studies also showed significantly decreased plasma concentrations of selenium in thalassemia major patients. Another important trace element is magnesium that plays an essential role in maintaining body's immune system as well as cardiovascular and musculoskeletal system found to be significantly higher in patients with beta thalassemia major as compared to controls [12]. Studies have shown that excess of iodine which is vital for the production of thyroxin and tri-iodothyronine may cause permanent hypothyroidism in beta thalassemia patients [22]. In addition, hypocalcaemia was found in beta thalassemia major patients than in controls [24, 25].

 On reviewing the studies published on antioxidant enzymes status in beta thalassemia, major patients also showed variable results. A significant increase in superoxide dismutase was found in beta thalassemia major patients [27] but another study showed no significant change in superoxide dismutase, catalase, and glutathione peroxidase with possible explanation proposed to be due to the presence of normal red cells owing to multiple blood transfusions [32]. Another important antioxidant enzyme glutathione reductase found to be significantly increased in beta thalassemia major patients may be due to the presence of enzyme regulated glutathione turnover system to overcome red cell damage. In one study, Glutathione peroxidase was found to be significantly increased [28] but opposite results with significantly decreased levels of glutathione peroxidase in another study [7]. The important antioxidant enzyme glutathione S-transferase was found to have genetic variations associated with patients on chelation therapy [31].

The administration of selective antioxidants along with essential trace elements and minerals in order to reduce the extent of oxidative damage and the related complications in beta thalassemia major still need further evaluation.
